# Investigation of Fire Protection Performance and Mechanical Properties of Thin-Ply Bio-Epoxy Composites

**DOI:** 10.3390/polym13050731

**Published:** 2021-02-27

**Authors:** Xiaoye Cong, Pooria Khalili, Chenkai Zhu, Saihua Li, Jingjing Li, Chris Rudd, Xiaoling Liu

**Affiliations:** 1 Faculty of Science and Engineering, University of Nottingham Ningbo China, Ningbo 315100, China; Xiaoye.Cong@nottingham.edu.cn (X.C.); zhyhome1989@163.com (C.Z.); Crystal.Li@nottingham.edu.cn (S.L.); 2 Swedish Centre for Resource Recovery, University of Borås, 501 90 Borås, Sweden; pooria.khalili@gmail.com; 3 National Engineering Technology Research Centre of Flame Retardant Material, School of Materials, Beijing Institute of Technology, 5 South Zhongguancun Street, Haidian District, Beijing 100081, China; 3120195559@bit.edu.cn; 4 James Cook University, Singapore 387380, Singapore; chris.rudd@jcu.edu.au

**Keywords:** bio-based epoxy laminate, thin-ply prepreg, flame retardant mat, mechanical properties, fire protection performance

## Abstract

Hybrid composites composed of bio-based thin-ply carbon fibre prepreg and flame-retardant mats (E20MI) have been produced to investigate the effects of laminate design on their fire protection performance and mechanical properties. These flame-retardant mats rely primarily on expandable graphite, mineral wool and glass fibre to generate a thermal barrier that releases incombustible gasses and protects the underlying material. A flame retardant (FR) mat is incorporated into the carbon fibre bio-based polymeric laminate and the relationship between the fire protection properties and mechanical properties is investigated. Hybrid composite laminates containing FR mats either at the exterior surfaces or embedded 2-plies deep have been tested by the limited oxygen index (LOI), vertical burning test and cone calorimetry. The addition of the surface or embedded E20MI flame retardant mats resulted in an improvement from a base line of 33.1% to 47.5% and 45.8%, respectively. All laminates passed the vertical burning test standard of FAR 25.853. Cone calorimeter data revealed an increase in the time to ignition (TTI) for the hybrid composites containing the FR mat, while the peak of heat release rate (PHRR) and total heat release (TTR) were greatly reduced. Furthermore, the maximum average rate of heat emission (MARHE) values indicated that both composites with flame retardant mats had achieved the requirements of EN 45545-2. However, the tensile strengths of laminates with surface or embedded flame-retardant mats were reduced from 1215.94 MPa to 885.92 MPa and 975.48 MPa, respectively. Similarly, the bending strength was reduced from 836.41 MPa to 767.03 MPa and 811.36 MPa, respectively.

## 1. Introduction

Due to good mechanical properties, chemical resistance properties and low density, carbon fibre reinforced epoxy resin composites play a key role in aerospace, automotive, sports, and energy fields. In recent decades, petroleum-based epoxy has received considerable attention, but with the increasing pressure of environmental protection and sustainability, new bio-based chemical raw material has been widely applied in epoxy resin manufacturing [1,2,3]. 

Rosin (C_19_H_29_COOH) is an abundant material that is derived from pine trees. More than 1 million metric tons of rosin is produced worldwide each year [4]. For the development of more sustainable thermosetting resins, Liu et al. synthesized two rosin-based imide curing agents and applied these to cure commercial epoxy resin. Compared to imide-diacid based curing agents, the two rosin-based curing agents showed higher tensile strength, tensile modulus and Tg [5]. They also tested rosin-based epoxy resin systems (resin and hardener) with rosin substitution and reported higher Tg, modulus, flexural properties, and better thermal stability as compared with conventional, fully petroleum-based epoxy resin systems [6]. Wang et al. reported that rosin-based maleopimaric acid imidoamine (MPAIA) as a curing agent displayed higher modulus and Tg when compared to those of the commercial curing agent [7].

In addition to mechanical performance, flammability remains a key concern for polymer and polymer composites in transportation applications. A variety of flame-retardant epoxies are available which rely on chemical flame-retardant agents or inorganic fillers [8,9,10,11]. Halogen-free flame-retardant agents are becoming increasingly common and, amongst these, expandable graphite (EG) plays an increasingly important role [12]. The flame retardance mechanism of EG shows that once-expanded graphite forms a thermal insulating barrier when burning, which not only prevents the underlying materials from combusting but also releases incombustible gases CO_2_ and H_2_O, which help to suppress flames [13]. Chiang et al. used EG grafted coupling agents (3-isocyanatopropyltriethoxysilane—IPTS) to manufacture composites, and the ensuing laminates demonstrated improved thermal stability and flame retardancy [14]. Similarly, Laachachi et al. found that adding EG to epoxy resin could significantly reduce its peak heat release rate (PHRR) value whilst reducing its ignition time [15]. Yang et al. combined EG with 9, 10-dihydro-9-oxa-10-phosphaphenanthrene-10-oxide (DOPO) and hexa-phenoxy-cyclotriphosphazene (HPCP) to manufacture epoxy composites with improved flame-retardant properties. The results demonstrated reduced PHRR values and a synergistic effect of the DOPO/EG and HPCP/EG hybrid additives [16]. He et al. used in-situ polymerization to synthesize ammonium polyphosphate (APP), which contained benzoxazine monomers, and combined this with EG before incorporating it into the composite. The result showed that the hybrid flame retardant agents promoted multifunctional performance and significantly improved flame retardancy and composite toughness [17]. Khalili et al. added EG powder as a filler and coating for natural fibre reinforced epoxy composites and reported fire protection properties that were influenced by the proportion of EG filler and coating. The EG filler resulted in a reduced heat transfer rate across the laminate at the cost of its mechanical properties [18]. 

In preliminary research, the authors of this work have investigated different flame retardant (FR) mats as a barrier layer to cover the surface of the composite and protect the composite upon exposure to flame. The results indicated that the fire-retardant mat was effective as a barrier layer and the general mechanism of protection was that the expanded graphite formed a protective char. It was also observed that the EG tended to peel away from the mat, which may pose a health issue [19]. 

In the current study, the FR mat was used as a hybrid reinforcement with thin prepreg plies. Two different lay-ups were studied, deriving from earlier investigations [19,20]. The effect of the z-wise local of the FR mat on the fire performance of laminates was investigated by thermogravimetric analysis (TGA), limit oxygen index (LOI), vertical burning and cone calorimeter tests. Mechanical testing was also performed to study the effect of the stacking sequences on the tensile and flexural behaviours of the laminates.

## 2. Materials and Methods

The rosin-based epoxy prepreg (AGMP-3600) with unidirectional Toray-T700-1200 carbon fibre (areal density: 75 g/m^2^, weight fraction: 60%) was provided by AVIC China (Beijing, China). This prepreg is manufactured by the two-step hot melt method [21] consisting of E-51 and solid phenolic mixing epoxy, including rosin-based anhydrides as the curing agent and two amino imidazole salt complexes as the latent catalyst [21]. Full details of the rosin-based epoxy system can be found in the previous literature [21,22], along with curing information based on FTIR, DSC and curing kinetics. The FTIR spectra showed that C=O groups decreased with increasing curing time. The cure kinetics study indicated that the resin system showed a broad peak under isothermal heating at 120 °C, and that when the curing temperature was above 120 °C, the resin system showed a high reaction activity [22].

The flame-retardant mat (E20MI) was produced by Technical Fibre Products Ltd. (UK) with specification found in Table 1. All samples were manufactured via compression moulding technology and then cured between heated platens at 120 °C and 5 MPa for 2 h. Zhang et al. [22] showed the viscosity of the resin system to range from 90 Pa∙s at 50 °C to 0.7 Pa∙s at 80 °C, with a gel point around the curing temperature (116 °C). This suggests that the resin system can achieve good infiltration during heating and is capable of producing good quality hybrid composites.

**Figure 1:**
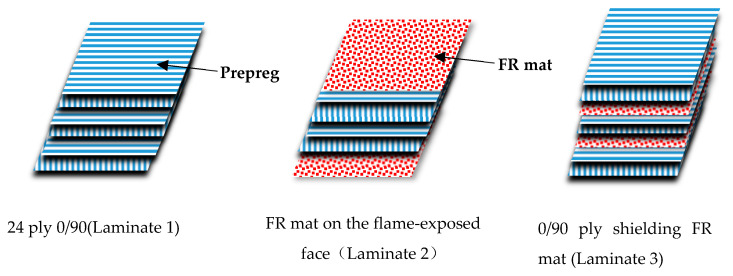
The different lay up of composite.

The stacking sequence was varied as follows:

(1) A 24 ply 0/90 control;

(2) The control laminate with one ply of FR mat on the flame-exposed face;

(3) A symmetric laminate with a single 0/90 ply on the outer face shielding an FR mat and a core of 22 plies of 0/90 prepreg (the detail lay-up is presented in Figure 1).

The tensile and flexural tests were carried out using an MTS E45 universal testing machine (MTS system corporation, Shenzhen China). The tensile strength and modulus were determined for batches of five specimens according to ISO 527 at a crosshead speed of 2 mm/min with the specimen dimension of 250 mm × 25 mm × 4 mm, and a gauge length of 50 mm. The bending properties were tested according to ISO 14125, also at a crosshead speed of 2 mm/min using 120 mm × 10 mm × 4 mm specimens with a span length of 64 mm.

A microscope (Zhejiang Yong xin Guangxue Co, Ltd, Ningbo, China) was used to confirm the thickness of the FR mat at various polished cross-sections.

The limiting oxygen index (LOI) test was performed in accordance with ISO 4589, using an oxygen index meter (TES Tech Instrument Co, Ltd., Suzhou, China), for 80 mm × 6.5 mm × 3 mm test specimens. The vertical burning test was measured using a test box supplied by the same manufacturer with 305 mm × 75 mm × 3mm specimens according to FAR 25.853. 

The thermal degradation of samples was carried out using a SDT Q600 instrument (TA instruments, New Castle, PA, USA). All samples were heated from 100 °C/min to 900 °C/min at a heating rate of 20 °C/min nitrogen environment (gas flow rate of 50 mL/min). The sample mass was approximately 10 mg. 

Cone calorimetry was performed using a Fire Testing Technology (West Sussex, UK) machine with a sample dimension of 100 mm × 100 mm × 3 mm, according to the ISO 5660-1. The heat flux was 50 KW/m^2^. 

## 3. Results and Discussion 

### 3.1. Morphology 

The resin content of the three different laminates is summarized in Table 2. The resin content was calculated using Equation (1). The resin content was seen to reduce with the addition of the FR mat because the (dry) FR mat absorbed excess resin from the prepreg and reduced the overall resin weight fraction.

Resin content = 1 − (W_f_ + W_m_)/W_c_ (1)

W_f_—carbon fibre weight; Wm—flame retardant mat weight; Wc—composite weight. 

**Figure 2:**
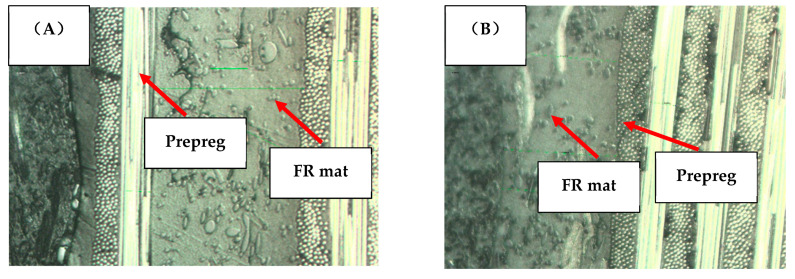
The cross-section of hybrid composites. (**A**) Laminate 2; (**B**) Laminate 3.

Transverse sections were studied by microscopy and the images are presented in Figure 2, revealing good bonding between the FR mat and prepreg layers in both laminates 2 and 3. 

### 3.2. LOI and Vertical Burning Test 

The LOI results are listed in Table 3. In general, the LOI values increased with the addition of the FR mat. As was expected from previous work, this improvement is due to the barrier formed by the expansion of graphite on heating and the release of incombustible gases [13,22]. The dense barrier prevented the combustible gas spreading into the flame and efficiently separated oxygen from burning matter [14]. After burning, the EG layer was protected by the mineral wool and glass fibre in the FR mat and prevented it from falling off, and finally achieved a good flame-retardant effect [19]. In the case of Laminate 2, the EG in the FR mat hindered the transfer of heat and oxygen into the pyrolysis region when the composite of Laminate 2 was ignited. However, for Laminate 3, the flame first ignited the resin of prepreg on the surface and then the flame and heat were transferred to the FR mat under prepreg. Then, the EG in the FR mat was activated and expanded as a thermal barrier to prevent the transfer of heat and oxygen to the composite [14,23]. This explains the slightly lower LOI value for Laminate 3 compared to Laminate 2.

**Figure 3:**
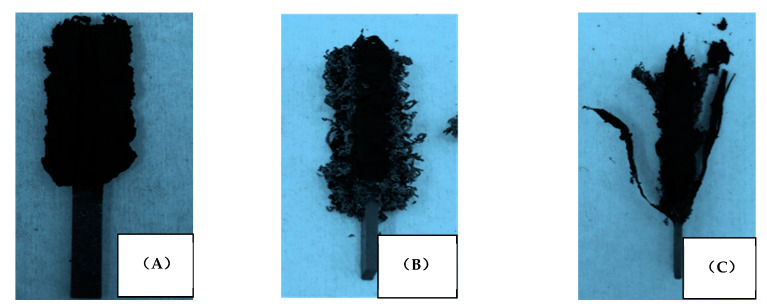
The images of the sample after LOI testing: (**A**) Laminate 1; (**B**) Laminate 2; (**C**) Laminate 3.

Images of different samples after the LOI test are shown in Figure 3. The control Laminate 1 (Figure 3A) exhibited a porous and expanded residual char on the surface, a residue from the combustion of epoxy resin. Laminate 2 presented a fluffy and expanded layer on the surface, which was formed by the EG and mineral fibre during combustion [19]. However, an interesting phenomenon was found on the surface of Laminate 3; it yielded a fibrous char with the surface prepreg separating from the laminate as the FR layer expanded, suggesting some measure of initial containment by the shielded FR ply.

**Figure 4:**
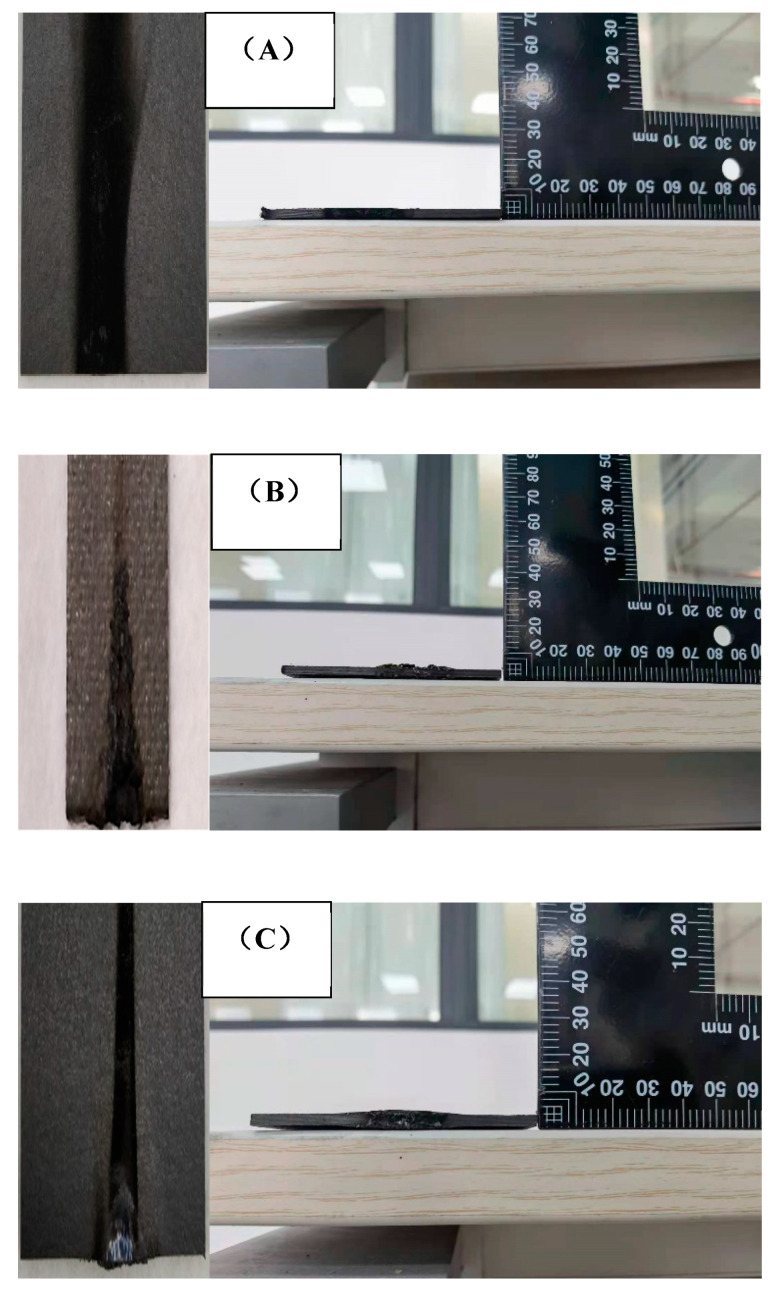
The images of the sample after the vertical burner test: (**A**) Laminate 1; (**B**) Laminate 2; (**C**) Laminate 3.

The results of vertical burner tests are shown in Table 3, including flame time and drip time. The flame time and drip time have been recognized as the important parameters in composite fire resistance properties. Dripping in particular can lead to fire growth, at last increasing the fire hazard. The total flame time of the control Laminate 1 was 22 s with no dripping. Both Laminate 2 and Laminate 3 were self-extinguished, showing no flame time and dripping, and subsequently the location of the FR layer made no difference. The images of composites after vertical burning testing are presented in Figure 4. Laminate 1 showed the phenomenon of charring, while both Laminate 2 and Laminate 3 demonstrated some char expansion, attributed to the EG action.

### 3.3. Cone Calorimeter Tests

Cone calorimeter results are listed in Table 4, with the time to ignition (TTI) of the control Laminate 1 being 39 s. This improved to 47 s for Laminate 2 and 45 s for Laminate 3. Clearly, the presence of the EG enhanced the thermal stability of Laminates 2 and 3 by acting as a physical barrier as well as releasing CO_2_ gas upon heating [19].

**Figure 5:**
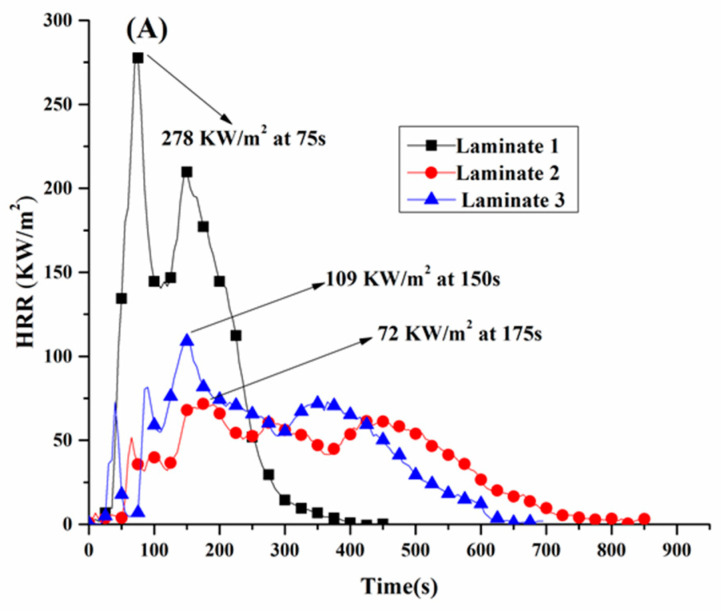

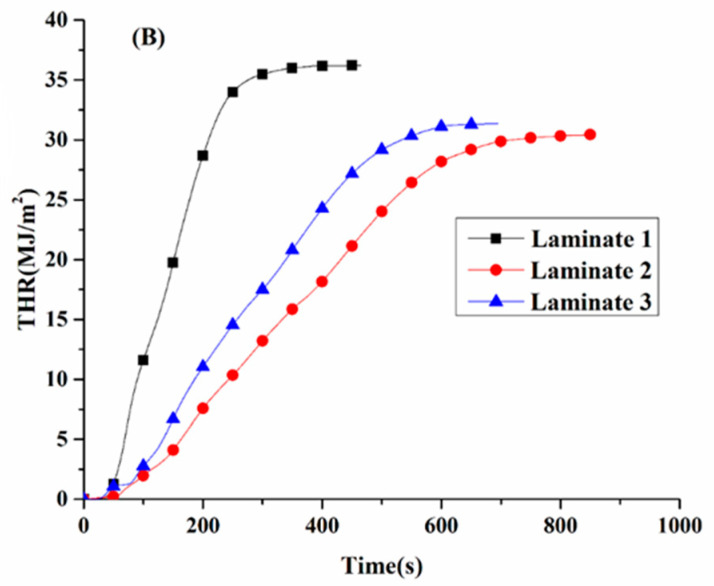
HRR (**A**) and total heat release (THR) (**B**) curves of different samples.

The peak heat release rate (PHRR) and total heat release (THR) shown in Figure 5 are also commonly applied to assess the fire safety of the material. These results showed that the HRR of Laminate 1 reached a peak around 278 kW/m^2^ at 75 s, but the PHRR of Laminates 2 and 3 decreased to 72 kW/m^2^ at 150 s and 109 kW/m^2^ at 175 s, respectively, representing reductions of 74% and 61%, respectively. 

The incorporation of FR mats obviously reduced the THR, which is simply the integra sum of all heat release. This is reflective of the calorific value of constituents and is relatively independent of the stacking sequence. The FR mat forms a carbonaceous layer which delays the complete combustion process, but there was a significant difference between Laminates 2 and 3. The smoke parameter is an important index in fire disasters that will influence human survival. The main source of smoke comes from the material under incomplete combustion [13]. 

The peak smoke production rate (PSPR) is shown in Table 4. The PSPR for Laminate 1 (control) was 0.11 m^2^/s. This decreased to 0.02 and 0.03 m^2^/s for Laminates 2 and 3, respectively. The barrier role of the FR mat clearly played a synergistic role in inhibiting the generation of smoke by protecting the otherwise flammable epoxy substrate.

The fire growth rate index (FIGRA) and the maximum average rate of heat emission (MARHE) are usually applied to evaluate the fire hazard of materials. The FIGRA values are relative measures of time available to escape from the fire scene [8], and are commonly calculated by Equation (2):

FIGRA = PHRR/t_PHRR_ (2)

t_PHRR_ means time to PHRR

FIGRA results for each laminate are listed in Table 4. Notably, Laminates 2 and 3 showed significant reductions in FIGRA values compared to the control sample. This confirms that the FR mat is effective in reducing the fire spread compared to the control laminate. The maximum average rate of heat emission (MARHE) is a proxy for the fire spreading tendency [18]. The addition of FR mats resulted in a reduction in MARHE values by 66.4% and 57.3% for Laminates 2 and 3, respectively. As such, both materials meet the EN45545 standard, which requires a MARHE value of lower than 90 KW/m^2^. This is attributed to the role of the EG in generating CO_2_ and H_2_O and forming a protective char layer, whilst the mineral and glass fibre stabilize the char and restrict the exchange of flammable volatiles and reduce heat transfer [19]. 

**Figure 6:**
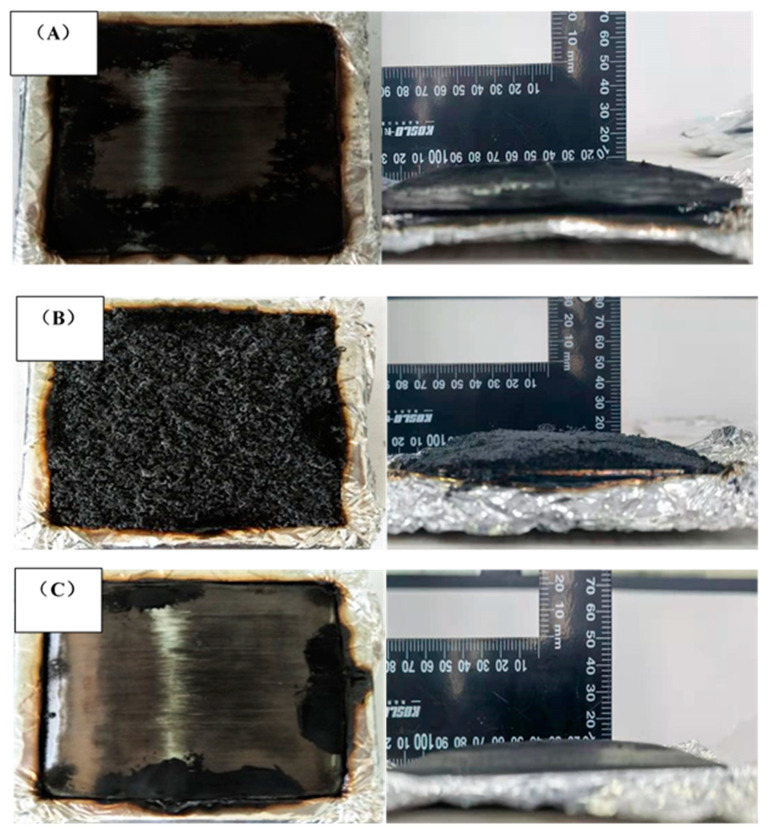
Images after cone calorimeter test: (**A**) Laminate 1; (**B**) Laminate 2; (**C**) Laminate 3.

**Figure 7:**
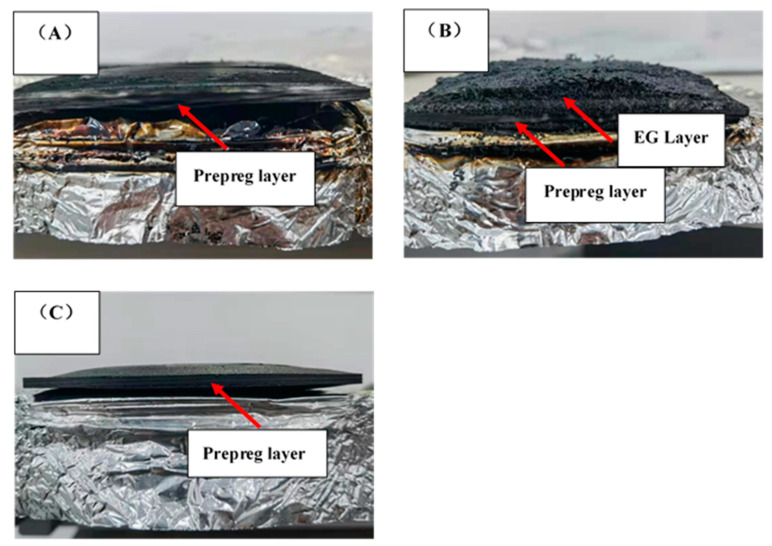
Fracture images after cone calorimeter tests: (**A**) Laminate 1; (**B**) Laminate 2; (**C**) Laminate 3.

The images of the samples after the cone calorimeter testing are shown in Figure 6 and Figure 7. Laminate 1 (Figure 6A) retained a smooth surface with residual carbon, but Laminates 2 (Figure 6B and Figure 7B) and 3 (Figure 6C and Figure 7C) underwent significant transverse expansion. However, the outward face of Laminate 3 remained smooth due to the prepreg facing ply Laminate 2, with its outer facing of the FR mat presenting an undulating surface of char. It is possible that the outer facing of prepreg in Laminate 3 would inhibit the potential exfoliation of airborne particles from the FR mat, which could pose health risks. For total smoke production (TSP), Laminate 1 had an indicative value of 13.2 m^2^, which was reduced to 3.6 m^2^ and 5.5 m^2^ for Laminate 2 and 3, respectively. Clearly, FR mat incorporation has the potential to reduce smoke emissions via the isolation of the bulk of the combustible material from the fire source [24].

### 3.4. Thermal Degradation Test 

Thermal stability was measured under a nitrogen atmosphere up to 900 °C, with the results outlined in Figure 6. Each of the samples showed a one-stage degradation progress, commencing at around 330 °C.

**Figure 8:**
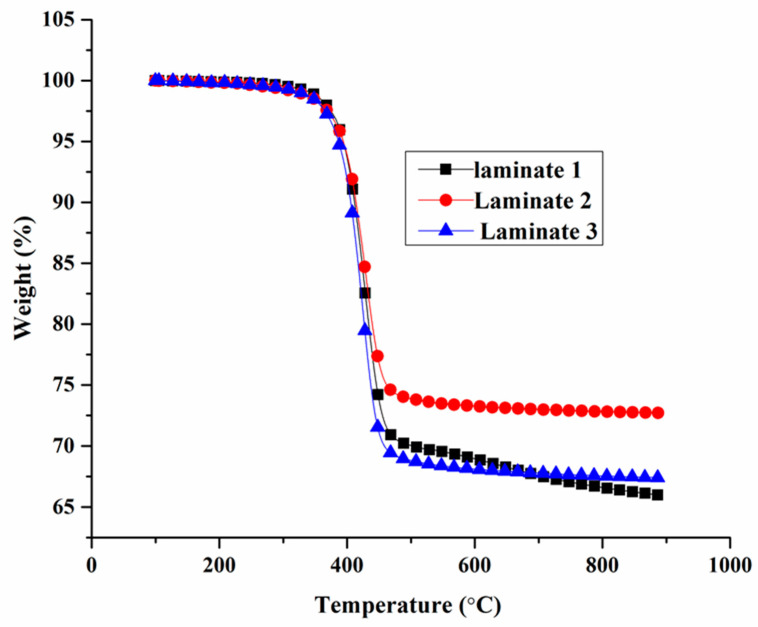
TG curve of different samples.

The char residual of Laminate 2 and Laminate 3 improved up to 72.77% and 67.53% compared to the control sample at 66.08%. The reason for this improvement was that the FR mat contained a high residual component, such as glass fibre and mineral fibre. It was thought that the EG in the FR mat showed cage effects, and it is the main reason for the improved char yield of the hybrid composite. Once the EG in the FR mat was heated to a certain degree, it would begin to expand and form a very thick layer of porous char. The thermal stability of the char layer was enough to separate the main body of the composite in order to delay the decomposition of hybrid composites [25]. The FR mat inserted into the composites showed a slightly higher thermal degradation temperature and mass residue, as indicated in Figure 8. The degradation temperature of FR samples improved from 425.00 °C to 427.66 °C and 430.11 °C, as obtained from derivative thermogravimetric results (Figure 8).

### 3.5. Mechanical Properties 

**Figure 9:**
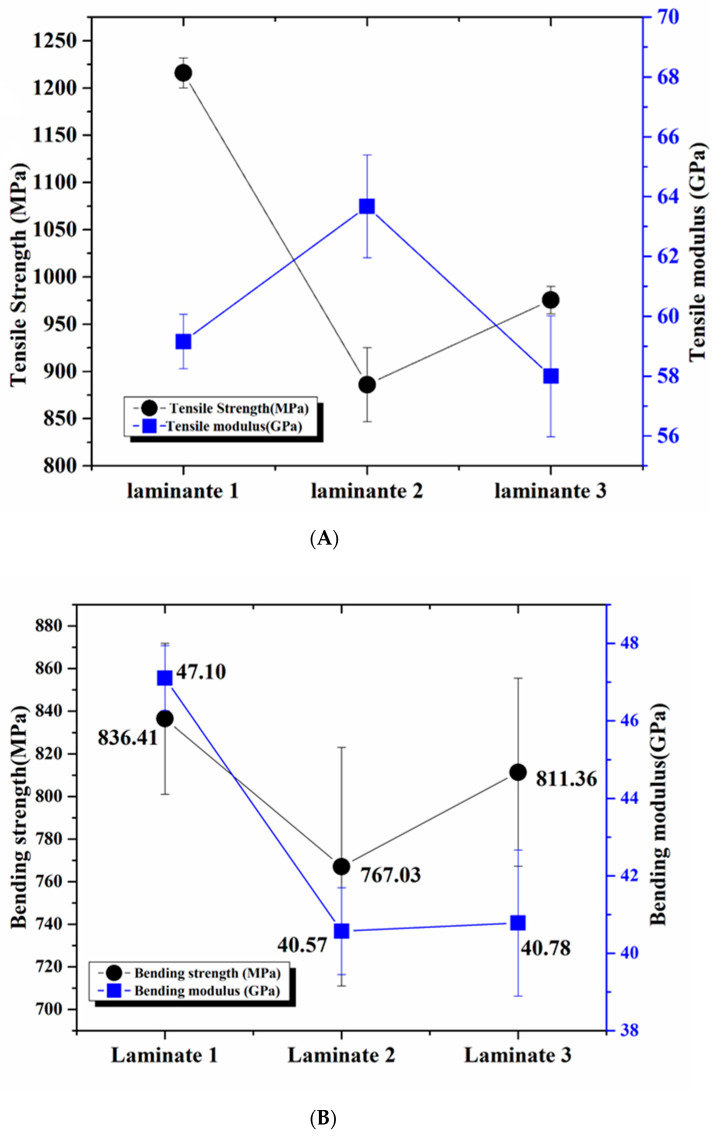
Tensile property (**A**) and bending property (**B**) curves of different samples.

In order to understand the mechanical properties of the different samples, all the corresponding data and curves of flexural and tensile testing are displayed in Figure 9. The tensile strength of the control sample was 1215.94 MPa, while the strengths of Laminate 2 and Laminate 3 reduced by 27.14% and 19.78% to 885.92 MPa and 975.48 MPa, respectively. It was found that the FR mat reduced the tensile strength, as random fibre and EG powder affected load transferred to carbon fibre, resulting in a significate strength reduction by 27.14% and 19.78%, respectively. However, compared to Laminate 3, Laminate 2 showed the highest reduction in tensile strength, which may be attributed to uniform stress in both the longitudinal and transverse direction of the fibre [26].

The theory of hybrid composite strength reduction can be recognized as the residual stress failure and progressive failure development. The residual stress failure is attributed to the difference of the coefficient of thermal expansion between different fibres, which becomes significant during processing. However, the influence of thermal expansion is expected to be small when the composites contain hybrid fibres [27,28]. Progressive failure is the most likely cause for the reduced tensile performance. As the strength of the FR mat was obviously lower than the prepreg, when the composites with the mat hybrid were loaded, the mat was damaged preferentially; the FR mat fails and the loads are transferred to the resin at the mat–laminate interface and finally to the nearby fibre (crack propagation) before complete failure. This is why the strength of the hybrid composite was lower than the pure prepreg composite. So, the crack development can be recognized as the main reason for the loading reduction [27,29,30]. Furthermore, the bending strength reduction can also be attributed to the same phenomenon.

Laminate 2, with the FR mat covering the surface of the composite, presented a lower strength than that of Laminate 3. The reason for this reduction was that the FR mat of Laminate 2 first failed and then transferred the failure to the prepreg, and at last, the hybrid composite was broken. The two layers of Laminate 3 were first broken and then transferred to the FR mat, but the FR mat showed lower strength than the prepreg. The flexural strength of the control sample was 836.41 GPa, whilst the composites of Laminate 2 and Laminate 3 were reduced to 767.03 and 811.36 MPa, respectively. It showed the same behaviour as tensile strength. The control sample showed the highest modulus of 47.10 GPa, and the composites of Laminate 2 and Laminate 3 were reduced to 40.57 GPa and 40.78 GPa, respectively. It was found that the pure composite had the highest bending resistance properties. 

In this study, an FR mat was included in the thin-ply bio-based prepreg for the first time, revealing that the FR mat location can influence both the fire protection results and the mechanical properties. Fadime Karaer Özmen et al. previously used red phosphorus and zinc borate and aluminium three hydrate as the flame-retardant agent to reduce the MARHE value, but the effect has not been as significant as in this work [31]. Jingjing Li et al. used the same FR mat on the outer surfaces of carbon fibre composites for a great reduction in the MARHE value to 7.08 kW/m^2^. However, the mechanical properties of the resulting composites also presented a reduction [19], and the observed peeling of the charred mats may pose health issues. Chenkai Zhu et al. have also used the same FR mat to protect honeycomb sandwich composite structures, with reduced MARHE values [32]. According to previous research, it is indicated that the FR mat with prepreg brings a new fire-resistant method, and achieves the requirement of EN45545-2.

## 4. Conclusions

In this study, the FR mat has been incorporated into two different stacking arrangements into carbon fibre reinforced polymer (CFRP) composites made from more sustainable resins. Specimens with surface FR mats (Laminate 2) and FR mats embedded 2-plies deep (Laminate 3) both demonstrated similar and significant improvements to the flame-resistant properties of a thin-ply CFRP laminate. The LOI results of Laminates 2 and 3 showed values of 47.5% and 45.8%, respectively, compared with 33.1% for the control. The vertical burning test results indicated that all the samples passed the test according to FAR25.853. However, peak heat release rates (PRHHs) were significantly reduced with the addition of the FR mats (from 278 kW/m^2^ to 75 kW/m^2^ and 172 kW/m^2^ for Laminates 2 and 3, respectively). The THR results reduced from 36 MJ/m^2^ to 30 MJ/m^2^ and 31 MJ/m^2^. The exact placement of the FR mats, either at the surface or beneath an orthongal pair of prepreg plies, did not appear to have a significant effect on the fire-retardant benefits of the hybrid laminates aside from the thermal stability. This was demonstrated by an increase in the thermal degradation temperature from 425.00 °C to 427.66 °C and 430.11 °C, and the mass residue increased from 66.08% to 67.53% and 72.77% after the addition of the FR mat. Lastly, mechanical testing revealed some reductions in the tensile and flexural properties of the hybrid laminates as a result of the FR mats.

## Figures and Tables

**Table 1:** Fire retardant mat (E20MI) specifications.

Product Name	Area Weight (Kg/m^3^)	Thickness (mm)	Typical Expansion Ration	Composition
E20MI	327	0.5	20:1	Mineral wool 20–50 wt.% Chopped glass fibre 4.5–40 wt.% Expanded Graphite 10–40 wt.% Binder 2.5–40 wt.%

**Table 2:** The formulation of composites and resin content.

Sample Code	Laminate 1	Laminate 2	Laminate 3
Resin content (wt.%)	36.3	31.1	33.7

**Table 3:** The values of the limited oxygen index (LOI) and vertical flame test.

Sample Code	LOI (%)	Vertical Burning		
		Flame Time(s)	Drip Flame Time(s)	FAR Requirement
Laminate 1 (Control)	33.1	22	0	Failed
Laminate 2	47.5	0	0	Pass
Laminate 3	45.8	0	0	Pass

**Table 4:** Cone calorimeter data of different samples.

Sample Code	Laminate 1 (Control)	Laminate 2	Laminate 3
TTI (s)	39	47	45
PHRR (kW/m^2^)	278	72	109
THR (MJ/m^2^)	36	30	31
FIGRA(kW/m^2^s)	3.71	0.73	0.41
FPI (s m^2^/KW)	0.14	0.65	0.23
TSP (m^2^)	13.2	3.6	5.5
PSPR (m^2^/s)	0.11	0.02	0.03
MARHE (KW/m^2^)	143	48	61

## Data Availability

The data presented in this study are available on request from the corresponding author.
